# The application of Caprini Risk Assessment Model in evaluation of deep vein thrombosis for patients with end-stage osteoarthritis before arthroplasty

**DOI:** 10.1186/s12891-022-05712-z

**Published:** 2022-08-12

**Authors:** Wei Sun, Dongmei Ai, Yao Yao, Kewei Ren, Jun Lu, Huiqing Sun, Xiaotao Wu, Qing Jiang

**Affiliations:** 1 Nanjing Medical University, Nanjing, China; 2 Department of Orthopedics, the Affiliated Jiangyin Hospital of Southeast University Medical College, Wuxi, China; 3 Department of Rehabilitation Medicine, Nanjing Drum Tower Hospital, The Affiliated Hospital of Nanjing University Medical School, Nanjing, China; 4 State Key Laboratory of Pharmaceutical Biotechnology, Division of Sports Medicine and Adult Reconstructive Surgery, Department of Orthopedic Surgery, Nanjing Drum Tower Hospital, The Affiliated Hospital of Nanjing University Medical School, Nanjing, China; 5 Laboratory for Bone and Joint Disease, Model Animal Research Center (MARC), Nanjing University, Nanjing, China; 6 The Spine Center, Department of Orthopedics, Zhong Da Hospital, School of Medicine, Southeast University, Nanjing, China

**Keywords:** Preoperative deep vein thrombosis, Caprini RAM, Knee arthroplasty

## Abstract

**Background:**

Deep vein thrombosis (DVT) was a fatal complication of knee arthroplasty. We had neglected the risk factors of preoperative DVT although patients undergoing knee arthroplasty were at high risk for VTE. This study was to determine the risk factors for preoperative DVT and application of Caprini Risk Assessment Model (RAM) in patients with end-stage knee osteoarthritis (OA).

**Methods:**

We retrospectively analyzed 1808 cases with end-stage knee OA undergoing primary knee arthroplasty from May 2015 to December 2020. Based on the results of ultrasonography in lower extremities, all patients were divided into non-DVT group and DVT group. Distribution of risk factors and risk levels were compared using χ^2^ test between two groups. Binary logistic regression analysis was used to determine the risk factors and relationship of risk levels and preoperative DVT.

**Results:**

The incidence of preoperative DVT was 5.53% (*n* = 100). Distribution of the study population by risk level was low, 4.09%; moderate, 23.95%; high, 66.98%; and highest 4.98%. Female (*P* = 0.002), age (*P* = 0.012), swollen legs (*P* = 0.035) and history of blood clots (*P* < 0.001) was correlated with preoperative DVT. Difference among four risk levels was significant (*P* = 0.007). Patients with highest risk level had statistically significant association with preoperative DVT (*P* = 0.005, OR = 2.93, 95%CI [1.375–6.246]).

**Conclusion:**

The incidence of preoperative DVT was 5.53% in end-stage knee OA patients. The gender (female) and age were independent risk factors for preoperative DVT. The risk group classification by Caprini RAM was significantly associated with preoperative DVT. The usage of Caprini RAM before knee arthroplasty may be beneficial for prophylaxis of DVT.

## Background

Venous thromboembolism (VTE), including deep vein thrombosis (DVT) and pulmonary embolism (PE), representing one of the major complications after knee arthroplasty, elevates morbidity and mortality during the perioperative period [1, 2]. It has been reported that the incidence of asymptomatic DVT after total knee arthroplasty (TKA) is 40–85% and the incidence of fatal PE is 0.87%-1.99% without drug intervention [3]. Numerous literatures focused on prevention and treatment for DVT after TKA [4–6]. Recently, several studies paid attention to the preoperative DVT in elderly patients with knee osteoarthritis (OA) who have severe disability [7, 8]. The incidence of preoperative DVT in TKA patients ranged from 6.7% to 17.9% [8–10]. Patients with DVT underwent surgical procedures like trauma or immobilization, DVT may extend, develop or even detach, resulting in PE, even death [7]. Given to the grave consequences of preoperative DVT, we screened all OA patients underwent TKA in our center with lower extremity venous ultrasound before operations to avoid the venture. However, preoperative ultrasound screen would increase patients’ medical costs and prolong physician’s operating time.

The Caprini score was originally proposed in the 1990s which provided an individualized and adequate VTE prophylaxis risk assessment model [11]. The score assigned points based on more than 20 risk factors taken from person’s history as well as their current health, in which a high score classifies a patient at a higher risk. The Caprini risk assessment model (RAM) has been verified by more than 100 clinical trials and 250 000 patients worldwide. Moreover, rational treatment regimens that depend on Caprini RAM were precisely accomplished [12]. In orthopedic surgery, especially joint arthroplasty and lower limb fractures, the Caprini RAM has been extensively used to evaluate the risk of postoperative DVT and provide the basis for precision therapy [13–15]. Nevertheless, the Caprini RAM has not been used to evaluate the quantitative risk until this study. The Caprini RAM also periodically updated based on evolution of the pathophysiology and risk factors of VTE [12, 16, 17].

End-stage knee OA patients were characterized with pain, old age and severe disability which lead to higher risk of DVT comparing to other diseases. Thus, our joint diseases center applied the Caprini RAM (2013version) to assess the risk of VTE in all hospitalized OA patients undergoing knee arthroplasty. The purpose of this study was to assess the effectiveness of the Caprini RAM in evaluating the risk of preoperative DVT and screen out the high risk factors in hospitalized OA patient undergoing knee arthroplasty.

## Materials and methods

With the approval of Ethics Committee in Clinical Institution of Nanjing Drum Tower Hospital, a retrospective review of inpatient database from Department of Sports Medicine and Adult Reconstructive Surgery was performed to identify patients underwent TKA or unilateral knee arthroplasty (UKA) from May 2015 to December 2020. Firstly, we included cases according to the following criteria: 1) Age > 18 years old; 2) Diagnosed as end-stage knee osteoarthritis; 3) Selective surgery, including TKA or UKA. Secondly, we excluded 178 cases with non-osteoarthritis underwent knee arthroplasty, 54 cases received non-arthroplasty procedure, such as arthroscopy, osteotomy, revision, infection debridement, etc., 13 cases who cannot receive ultrasound screening for thrombosis diagnosing due to various conditions and 23 cases with incomplete data information retention and cannot be retrospectively analyzed for various reasons. Finally, a total of 1808 cases were enrolled. All cases were divided into non-DVT group (*n* = 1708 cases) and DVT group (*n* = 100 cases), according to the results of ultrasonography for DVT in bilateral lower extremities. Retrospective Caprini RAM was used for preoperative DVT risk assessment. All methods in this study were in accordance with the relevant regulations and guidelines.

### Preoperative DVT assessment

Each patient received preoperative evaluation for DVT within 3 days before surgery. A bedside ultrasound (SonoSite M-Turbo) was utilized on bilateral lower extremities to preclude DVT by a highly skilled sonographer. The following veins including bilateral common iliac vein, femoral vein, superficial femoral vein, popliteal vein, peroneal vein, anterior tibial vein, posterior tibial vein and muscular veins were mainly examined. The criteria for diagnosing DVT were: 1) Failure of full compressibility of venous segment; 2) Absence or abnormal flow on color Doppler and spectral analysis.

### Caprini risk assessment

Caprini RAM (2013 Version) was applied in this study [12]. The cumulative risk score and risk level of Caprini risk assessment were retrospectively conducted, based on the inpatient database from Department of Sports Medicine and Adult Reconstructive Surgery. Body mass index (BMI) was automatically calculated by system with documented height (m) and weight (kg) as follows: BMI = weight divided by the square of your height. Risk factors for each patient were scored by the same professional with in-depth training of Caprini RAM and checked by an experienced physician. Each risk factor has different score (Table 3). Then, summed all risk factors scores to determine the risk level. Total score of 0–1, 2, 3–4 and ≥ 5 are defined as low-risk level, moderate-risk level, high-risk level and highest-risk level respectively. The reliability of this kind of retrospective scoring method has been reported in previous research [18].

### Statistical analysis

All the data were analyzed with the use of SPSS statistical software 22.0 (USA). Mean ( ±) standard deviation (x ± s) was calculated to describe continuous variables (age, height, weight and BMI) by independent sample t-test. Differences in the percentage of DVT incidence in two groups were compared using a χ^2^ test. The distribution of the incidence rate of DVT by risk level and the significance of differences was reported using χ^2^ test. Binary logistic regression analysis was used to determine the risk factors and relationship of risk levels and preoperative DVT. BMI was excluded from the final model since its coefficient estimates was insignificant contributing to the model fit. For risk level logistic regression analysis, risk level was analyzed as an ordinal categorical variable. Due to the distribution of preoperative DVT in low risk level is 0, low and moderate risk level were combined and coded 1, high and highest risk level were coded 2 and 3 respectively. *P* < 0.05 was considered as significant value.

## Results

### Information of clinical data

In this retrospective study, a total of 1808 inpatients underwent knee arthroplasty were retrospectively enrolled in this study, with 80.75% cases undergoing TKA surgery, and 19.25% cases undergoing UKA surgery. There are 1421 females and 387 males. The number of females in DVT group is markedly higher than that in non-DVT group (*P* = 0.002). Age in DVT group was significantly older than that in non-DVT group (71.67 ± 6.75 vs. 68.22 ± 7.65, *P* < 0.001). There is no significant difference in BMI between DVT group and non-DVT group (26.55 ± 3.79 vs. 27.05 ± 3.85, *P* = 0.21). The binary logistic regression showed that gender-female and age were independent risk factors for preoperative DVT (*P* = 0.002, OR = 2.877, 95%CI = 1.473- 5.618; *P* < 0.001, OR = 1,071, 95%CI = 1.041–1.103). (Table 1).

**Table 1:** Demographic characteristics of Non-DVT group and DVT group

Characteristic	Non-DVT (*n* = 1708)	DVT (*n* = 100)	t	*P* value^‡^	OR [95% CI]
Gender^a^					
Female	1331, (77.93%)	90, (90.00%)	-	0.002*	2.877 [1.473- 5.618]
Male	377, (22.07%)	10, (10.00%)	-		
Age (yrs)	68.22 ± 7.65	71.67 ± 6.75	-4.408	< 0.001*	1.071 [1.041–1.103]
Height (cm)	159.15 ± 7.42	157.77 ± 7.07	1.811	0.070	
Weight (kg)	68.57 ± 11.08	66.25 ± 11.30	2.037	0.042*	
BMI (kg/m^2^)	27.05 ± 3.85	26.55 ± 3.79	1.254	0.210	-

*DVT* Deep vein thrombosis, *BMI* Body mass index, *yrs* years

^a^The values are given as the number of patients, with the percentage in parentheses. *P* values was calculated using χ^2^ test

^‡^ *P* value of χ^2^ test

^*^ *P* < 0.05 was considered statistically significant

### Incidence and distribution of preoperative DVT

100 patients (5.53%) were diagnosed as DVT before knee arthroplasty. Among them, 12 cases were found thrombosis in bilateral legs; 48 cases had thrombosis in the affected leg (operated leg), and 40 cases in the contralateral leg. For patients with proximal thrombosis, 1 case occurred in iliac vein, 2 cases occurred in femoral vein, 2 cases occurred in popliteal vein and 1 case had mixed thrombosis in both popliteal and muscular veins. While, for patients with distal thrombosis, majority of cases (*n* = 93) occurred in muscular veins, and 1 case occurred in both muscular and peroneal veins (Table 2).

**Table 2:** Locations of DVT

Locations	Number of Cases
Proximal DVT	
Iliac	1
Femoral	2
Popliteal	2
Popliteal + Muscular	1
Distal DVT	
Muscular	93
Muscular + Peroneal	1
Total	100

*DVT* Deep venous thrombosis

### Comparison of cumulative score and risk factors between non-DVT and DVT groups

**Fig. 1:**
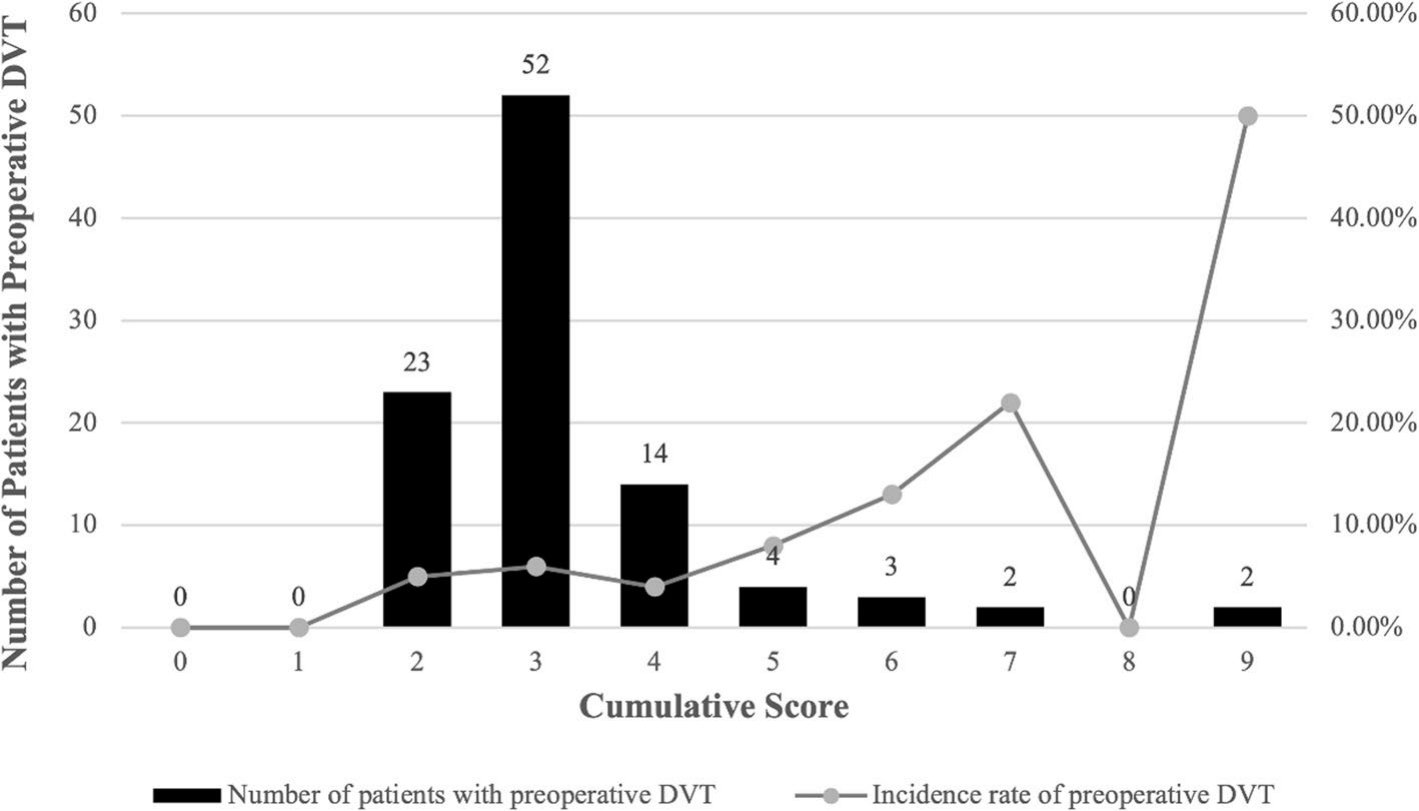
Distribution of cumulative Caprini score

The range of cumulative score of the study population is from 0 to 9. Distribution by each cumulative score was 0 (0.11%), 1 (3.98%), 2 (23.95%), 3 (49.72%), 4 (17.26%), 5 (2.93%), 6 (1.33%), 7 (0.50%), 8 (0%) and 9 (0.22%) respectively. The detailed distribution of cases and incidence rate of preoperative DVT is illustrated in Fig. 1. The growth of preoperative DVT appears to accelerate according to their cumulative risk score, especially significant for cumulative risk scores of 6–9. Meanwhile, the mean of cumulative score in DVT group is mildly higher than non-DVT group (3.28 ± 1.33 vs. 2.96 ± 0.96,* P* = 0.022) (Table 2).

Among 37 risk factors listed in the Caprini risk assessment mode, age (*P* = 0.012), swollen legs (*P* = 0.035) and history of blood clots (*P* < 0.001) were associated significantly with increased risk of DVT incidence before knee arthroplasty (Table 3). The incidence rate increased with age significantly (0.00% in ≤ 40 years, 2.43% in 41-60 years, 5.26% in 61–74 years and 8.31% in ≥ 75 years, *P* = 0.012).

**Table 3:** Comparison of caprini risk factors between non-DVT group and DVT group

Relative Risk Factors		Risk Score	Non-DVT (n, %)	DVT (n, %)	*P* value
Age^†^	≤ 40 yrs	-	5, (0.29)	0, (0.00)	0.012*
	41–60 yrs	1	241, (14.11)	6, (6.00)	
	61–74 yrs	2	1098, (64.29)	61, (61.00)	
	≥ 75 yrs	3	364, (21.31)	33, (33.00)	
Visible varicose veins		1	45, (2.63)	2, (2.00)	0.698
History of inflammatory bowel disease		1	2, (0.12)	0, (0.00)	0.732
Swollen legs (current)		1	2, (0.12)	1, (1.00)	0.035*
BMI, 25–40 kg/m^2^		1	1189, (69.61)	60, (60.00)	0.043*
Lung disease (eg, emphysema or COPD)		1	17, (1.00)	0, (0.00)	0.316
Other risk factors (< 1 mo)	BMI, ≥ 40 kg/m^2^	1	6, (0.35)	0, (0.00)	0.553
	Smoking	1	71, (4.16)	2, (2.00)	0.287
	Diabetes requiring insulin	1	45, (2.63)	4, (4.00)	0.414
For women only	Current use of birth control or hormone replacement therapy(HRT)	1	4, (0.23)	0, (0.00)	0.628
Current or past malignancies		2	57, (3.34)	3, (3.00)	0.855
History of blood clots		3	9, (0.53)	7, (7.00)	< 0.001*
Total score^‡^		-	2.96 ± 0.96	3.28 ± 1.33	0.022*

*DVT* Deep venous thrombosis, *BMI* Body mass index, *COPD* Chronic obstructive pulmonary disease, *yrs* years, *mo* month

^†^χ^2^test to analysis the incidence of perioperative DVT in different age groups

^‡^ The values are given as the mean ± standard deviation

^*^ *P *< 0.05 was considered statistically significant

### Comparison of risk levels between non-DVT and DVT groups

**Fig. 2:**
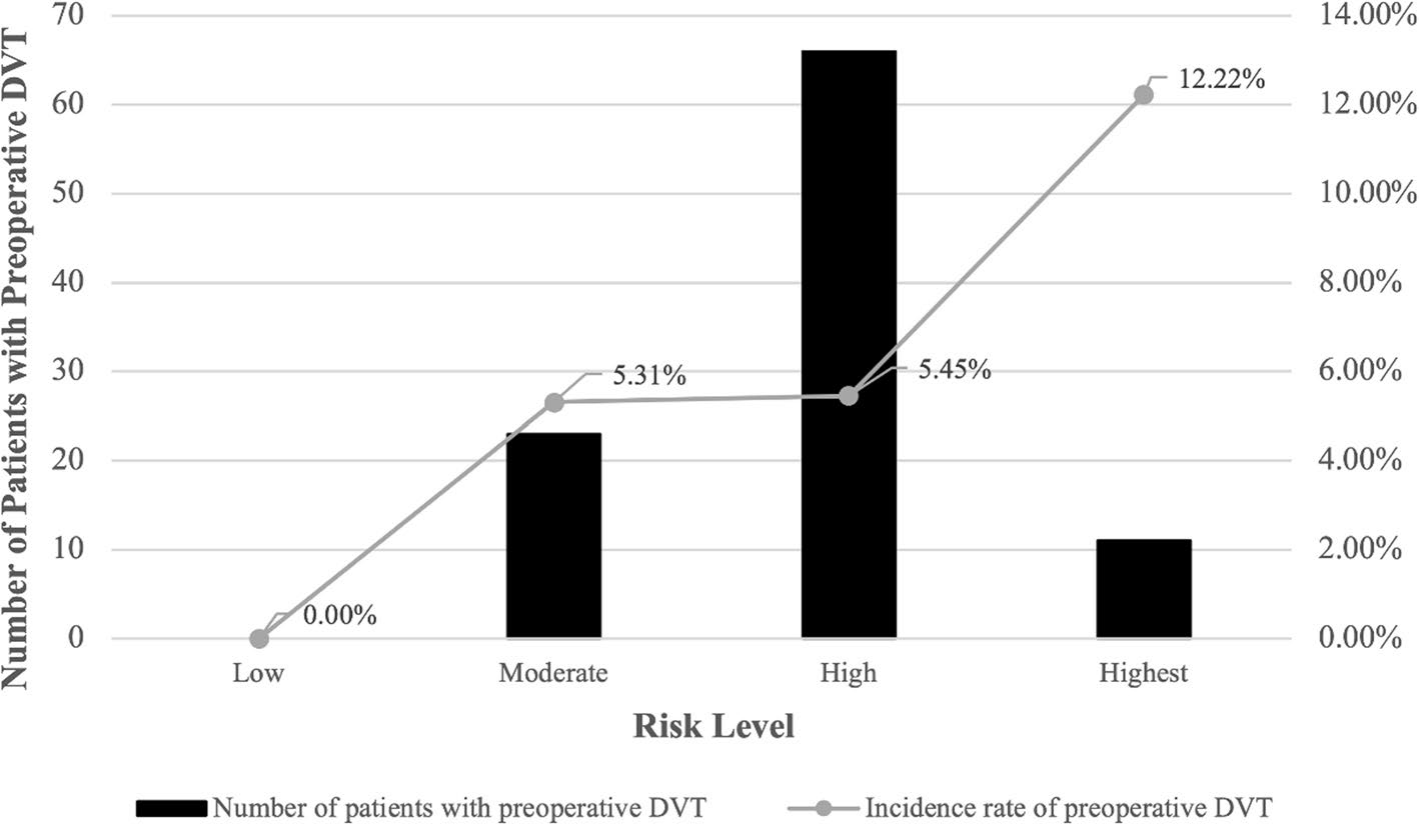
Incidence rate of preoperative DVT by risk level of Caprini RAM

The distribution by risk levels for these 1808 cases was low (4.09%), moderate (23.95%), high (66.98%) and highest (4.98%). Figure 2 demonstrated the distribution of preoperative DVT in different risk levels. The incidence of preoperative DVT was correlated with increase of risk level. In the highest risk level, 12.22% cases acquired DVT; patients with high and moderate risk level, 5.45%; and patients with low risk, 0%. The difference among 4 risk levels was statistically significant (*P* = 0.07) (Table 4). Additionally, in the low risk group, the preoperative DVT rate were significant lower than other groups (*P* = 0.034), while that in the highest risk group were significant higher than others (*P* = 0,004).

**Table 4:** Comparison of caprini risk level between non-DVT and DVT groups

Risk Levels	**Non-DVT(** ***n*** **, %)**	**DVT** **(** ***n*** **, %)**	**Incidence Rate of Preoperative DVT**	***P*** ** value**
Low	74, (4.33)	0, (0.00)	0.00%	0.007*
Moderate	410, (24.00)	23, (23.00)	5.31%	
High	1145, (67.04)	66, (66.00)	5.45%	
Highest	79, (4.63)	11, (11.00)	12.22%	

*DVT* Deep venous thrombosis；^*^ *P *< 0.05 was considered statistically significant.

The binary logistic regression in Table 5 showed that compared to low and moderate risk levels, cases in high risk level had mildly higher risk (1.213 times) of preoperative DVT (*p* = 0.436, OR-1.213, 95%CI = 0.746–1.973). While, cases in highest had a significantly higher risk (2.93 times) acquiring DVT before knee arthroplasty (*P* = 0.005, OR = 2.93, 95%CI = 1.375–6.246).

**Table 5:** Binary regression analysis of risk levels

Risk Levels	***P*** ** value**	**OR**	**95% CI**
Low and moderate	0.017		-
High	0.436	1.213	0.746–1.973
Highest	0.005	2.93	1.375–6.246

*OR* Odds ratio, *CI* Confidence interval

## Discussion

Knee arthroplasty has been proven to be safe, cost effective, and widely undertaken for improving quality of life in patients with end-stage knee osteoarthritis [19]. Age, obesity, metabolic syndrome, tourniquet use and other risk factors have been found to be associated with DVT after TKA [4, 20, 21]. However, patients undergoing TKA were at high risk for VTE [7]. End-stage OA patients characterized with pain and high age could increase the risk for DVT [22, 23]. To survey the incidence of surgery-related DVT, the incidence of patients showing DVT before surgery must be subtracted from the total of the incidences before and after surgery. In present study, the incidence of preoperative DVT in end-stage knee OA patient underwent knee arthroplasty was 5.53% which was similar to the preceding literatures [7, 8]. Compared to the 17.9% incidence reported by Wakabayashi et al. [9], our incidence of preoperative DVT was significantly lower. However, it should be noted that subjects with rheumatoid arthritis (RA) accounted for 34% of the sample size in their study, as we know, patients with RA were more prone to DVT comparing to OA patients due to chronical inflammation [24, 25]. In this study, the Caprini RAM (2013 Version) was used to conduct preoperative VTE score and risk grade classification for patients with end-stage OA. The Caprini RAM scores in DVT group were significantly higher than those in non-DVT group (3.28 ± 1.33vs2.96 ± 0.96, *P* = 0.022). Besides, we studied the incidence of preoperative DVT in patients with different Caprini score and levels. The preoperative DVT rates increased with the improvement of Caprini RAM score and risk level. Furthermore, the preoperative DVT incidence was significantly correlated with Caprini RAM risk level (*P* = 0.007). The age was an independent risk factor for preoperative DVT (*p* < 0.001) in Caprini RAM. Different age stratification got different score. After age stratification, the correlation was consistent (*p* = 0.012). Among 37 risk factors listed in the Caprini risk assessment mode, age (*P* = 0.012), swollen legs (*P* = 0.035) and history of blood clots (*P* < 0.001) were associated significantly with increased risk of DVT incidence before knee arthroplasty. Commonly, elderly patients were more vulnerable to have high blood viscosity, vascular sclerosis, and poor venous valve function, which lead to a high incidence of DVT [26]. As we had known, unilateral limb swelling would raise the suspicion of DVT [27]. In our study, 9 cases (0.53%) with history of blood clots in non-DVT group and 7 cases (7%) in DVT group, history of blood clots was significantly associated with DVT. In our study, the risk of preoperative DVT in the low risk group was significantly lower than that in other groups (*P* = 0.034), while that in the highest risk group was significantly higher than others (*P* = 0.004). These results all verified the validity of Caprini RAM to evaluate preoperative DVT risk in end-stage OA patients.

As far as we know, proximal DVT was more prone to cause adverse events (potentially fatal) than distal DVT [28, 29]. In our study, a total of 6 cases (6%) were found to be proximal DVT and positive treatment measures were taken, none of them had serious consequences. Although gender was not included in Caprini RAM, our research addressed that female was an independent risk factor for preoperative DVT, indicating that clinical workers should probably pay more attention to the DVT occurrence for female patients before knee arthroplasty. In previous studies [28, 29], gender (female) seemed to be a risk factor for DVT, however, the association had no statistical significance due to their limited sample sizes. It’s worth noting that our sample size was more than 10 times than theirs, which providing a more solid support for our study result and suggestion for clinical application.

The Caprini RAM had been evaluated to be useful not only for surgical patients but also in medical patients [30–32]. In this study, Caprini RAM was used for preoperative DVT evaluation in the end-stage knee OA patients undergoing knee arthroplasty for the first time. This work verified the effective function of Caprini RAM in preoperative DVT risk screening for patients with TKA or UKA. Nonetheless, this study has limitations inherent to retrospective single-center study. There are still other confounders not introduced into the analysis due to the retrospective nature of this study. Also, we only tested for preoperative DVT by using Doppler ultrasonic, which may lead to missed subsequent DVT. However, previous studies had reported that Doppler ultrasonic could replace venography as a screening tool for DVT. Moreover, all ultrasound screening in our study was performed by a greatly skilled physician who has experience of thousands cases of thrombus screening. Meanwhile, it is one-sided to analyze the incidence of preoperative DVT only via Caprini RAM. In the future, we can develop a more comprehensive and accurate evaluation system for preoperative DVT, combining physical questionnaires with routine examination, such as blood examination and other relevant data.

## Conclusion

In conclusion, this study evaluated the incidence of preoperative DVT and assessed risk factors by Caprini RAM in end-stage OA patients underwent knee arthroplasty. preoperative DVT was diagnosed in 100 of 1808 (5.33%) patients overall. The Caprini RAM had a significant function in preoperative DVT screening. We found the gender (female) and age were independent risk factors for preoperative DVT in end-stage knee OA patient underwent knee arthroplasty. The risk group classification by Caprini RAM was significantly associated with preoperative DVT. The usage of Caprini RAM before knee arthroplasty may be beneficial for prophylaxis of DVT. We suggested the low-risk group classification by Caprini RAM could be exempted from color doppler ultrasound examination while ultrasound screening was still recommended for the other risk grades, especially for the highest risk grade.

## Data Availability

The datasets used and/or analyzed during the study are available from the corresponding author on reasonable request.

## Abbreviations

BMI: Body mass index
CI: Confidence interval
DVT: Deep vein thrombosis
OA: Osteoarthritis
OR: Odd ratio
PE: Pulmonary embolism
RAM: Risk assessment model
TKA: Total knee arthroplasty
UKA: Unilateral knee arthroplasty
VTE: Venous thromboembolism
